# Evaluation of Midlife Educational Attainment Among Attendees of a Comprehensive Early Childhood Education Program in the Context of Early Adverse Childhood Experiences

**DOI:** 10.1001/jamanetworkopen.2023.19372

**Published:** 2023-06-22

**Authors:** Alison Giovanelli, Christina F. Mondi, Arthur J. Reynolds, Suh-Ruu Ou

**Affiliations:** 1 Division of Adolescent and Young Adult Medicine, Department of Pediatrics, University of California, San Francisco; 2 Brazelton Touchpoints Center, Division of Developmental Medicine, Boston Children’s Hospital, Harvard Medical School, Boston, Massachusetts; 3 Human Capital Research Collaborative, Institute of Child Development, University of Minnesota, Minneapolis

## Abstract

**Importance:**

Educational attainment is a key social determinant of health and can be particularly consequential for racial and ethnic minority populations. Although the consequences of adverse childhood experiences (ACEs) are well established, there is little research on protective factors and policy-relevant strategies to mitigate ACE-related inequities.

**Objective:**

To examine associations between early ACEs, comprehensive early intervention, and midlife educational attainment in a cohort of predominantly Black participants.

**Design, Setting, and Participants:**

The Chicago Longitudinal Study is a prospective cohort study of Black and Latinx children from Chicago, Illinois. The intervention group included 989 children entering the Child-Parent Center (CPC) preschool Early Childhood Education (ECE) program in the 1980s. The comparison group comprised 550 children participating in usual early childhood services. All participants were followed up for 30 years after the end of the intervention. Analyses were conducted from July 1 to September 1, 2022.

**Intervention:**

Attendance at the CPC preschool ECE program.

**Main Outcomes and Measures:**

A standard battery of early childhood ACEs (conventional ACEs), a set of early childhood ACEs more commonly associated with high-poverty contexts (expanded ACEs), and educational attainment at 35 years of age were measured from self-report and administrative records.

**Results:**

The original Chicago Longitudinal Study sample comprised 1539 participants (1430 Black participants [92.9%]; 774 female participants [50.3%]). Data on educational attainment and ACEs were available for 1083 of 1467 living participants (73.8%). Participants in the present study (1013 Black participants [93.5%]; 594 female participants [54.9%]) were a mean (SD) age of 35.1 (0.3) years at completion of the midlife survey. For the comparison group but not the CPC intervention group, having 1 or more conventional or expanded ACEs in early childhood was associated with fewer years of education (β = −0.64; 95% CI, −1.02 to −0.26), reduced likelihood of attaining a bachelor’s degree or higher (odds ratio, 0.26; 95% CI, 0.09-0.70), and reduced likelihood of attaining an associate’s degree or higher (odds ratio, 0.26; 95% CI, 0.11-0.62) after adjusting for covariates. Moderation analyses indicated that CPC participants with either conventional or expanded ACEs in early childhood attained a bachelor’s degree or higher and an associate’s degree or higher at rates similar to CPC participants without early ACEs (15.4% vs 13.6% for bachelor’s degree or higher; 22.4% vs 19.9% for associate’s degree or higher). Conversely, comparison group participants with early ACEs had significantly lower rates of educational attainment than their counterparts without ACEs (3.7% vs 12.1% for bachelor’s degree or higher; 5.6% vs 17.1% for associate’s degree or higher).

**Conclusions:**

This cohort study suggests that early ACEs were associated with reduced educational attainment for the comparison group but not for the group participating in the CPC comprehensive early intervention. These results build on research suggesting that youths at higher risk can benefit most from intervention and support ECE as a tool for reducing ACE-related disparities.

## Key Points

**Question:**

Is exposure to early adverse childhood experiences (ACEs) associated with reduced midlife educational attainment, and if so, did participation in the Child-Parent Center (CPC) comprehensive Early Childhood Education (ECE) program attenuate these associations?

**Findings:**

In this cohort study of 1083 participants, early ACEs were significantly associated with reduced midlife educational attainment for the comparison group, but not for the CPC early intervention group. The CPC preschool program appeared to compensate for associations between ACEs and educational attainment; CPC attendees with early ACEs had similar educational trajectories to those of CPC attendees without early ACEs.

**Meaning:**

This study suggests that comprehensive ECE can be a scalable approach for promoting long-term educational success after early experiences of ACEs.

## Teaser

This cohort study examines associations between early adverse childhood experiences (ACEs), comprehensive early intervention, and midlife educational attainment in a cohort of predominately Black participants.

## Introduction

Research on adverse childhood experiences (ACEs; abuse, neglect, and household dysfunction) has focused extensively on associations of ACEs with long-term mental and physical health.^1,2,3^ More recently, ACEs have been associated with academic performance^4,5,6^ and high school graduation.^7,8^ Educational attainment, in turn, is associated with mental and physical well-being, primarily through economic mobility and stability,^9,10,11,12,13,14^ and can be particularly consequential for racial and ethnic minority populations.^15^

### Comprehensive Early Childhood Education for Children in Poverty

Comprehensive Early Childhood Education (ECE) programs—programs providing educational, family, and health care services to young children and their families—are effective preventive interventions for children in disadvantaged environments,^16,17,18,19^ affecting broad domains of well-being and providing economic returns on investment.^19,20^ Longitudinal research, including investigations with the present sample,^21,22^ demonstrates marked associations between ECE and educational attainment.^18^ Moreover, ECE has also been found to be most beneficial for children at highest risk, such as those with a history of maltreatment or with greater relative sociodemographic disadvantage.^21,23^ Considering this evidence, ECE may also be differentially beneficial for children who are high risk due to early ACE exposure and could therefore be an effective intervention for mitigating the association of ACEs with educational attainment.

### Childhood Poverty and ACEs

Although ACEs are associated with mental and physical illness and shortened lifespan even in well-resourced populations,^1,2,3^ poverty can amplify both the incidence and consequences of ACEs.^24,25,26^ Black people in the US are more likely than White people to experience both poverty and ACEs.^27,28^ The full implications of this are unknown; although ACE studies have increasingly been conducted with socioeconomically and racially and ethnically diverse samples,^29,30,31,32^ large-scale longitudinal research on ACEs in low-income Black populations remains relatively scarce.

Despite this limitation, cross-sectional data suggest that in addition to experiencing increased rates of conventional ACEs (eg, those included in the original ACE studies [ie, abuse, neglect, and household dysfunction]), Black and low-income populations are also more likely to be exposed to sociocontextual ACEs, such as neighborhood violence and out-of-home placement.^33,34,35,36^ Such experiences, termed *expanded ACEs*,^30,37,38,39^ can occur in the absence of conventional ACEs^40,41^ and have been associated with well-being above and beyond the effects of co-occurring conventional ACEs.^32,37^ Broadening the definition of ACEs could provide a more accurate picture of adversity in underserved populations and a more complete understanding of the mechanisms through which adversity is associated with poor outcomes,^37,40^ but research on the consequences of a range of ACEs relevant to diverse populations is needed.

### ACEs in the Present Sample

The present sample is in early midlife. When these participants were 24 years of age, research suggested that early childhood ACEs had lasting consequences for multiple dimensions of their well-being.^7^ Participation in the ECE program promoted resilience through later experiences and student motivation, particularly for participants with a history of ACEs.^42^ At the same time, participants with early ACEs were less than half as likely to graduate from high school than participants without early ACEs.^7^ Because few participants had earned a postsecondary degree by 24 years of age, associations between ACEs and completion of postsecondary education could not be examined. By 35 years of age, rates of participants attaining higher education had increased, presenting an opportunity to more thoroughly assess associations between ACEs and postsecondary educational attainment.

In the present study, we aim to evaluate whether ACEs and ECE intersect with regard to higher education. To our knowledge, this is the first study to examine the association of ACEs with educational attainment in a longitudinal sample of majority Black participants, as well as the first to investigate the potential of ECE to buffer ACE-exposed youths from the deleterious effects of ACEs. Moreover, the potential of the focal ECE program to benefit children with ACEs is of particular interest as it is currently operating at scale across the Midwest. We hypothesize that (1) exposure to any conventional or expanded ACEs in early childhood will be associated with reduced educational attainment in a low-income cohort of primarily Black participants in early midlife and (2) a comprehensive ECE preventive intervention will compensate for hypothesized associations between ACEs and educational attainment.

## Methods

### Study Design

The Chicago Longitudinal Study is a prospective, matched-group multisite study investigating the association of early experiences and the Child-Parent Center (CPC) preschool through third grade intervention^22^ with life-course well-being for 1539 participants who were born in high-poverty Chicago, Illinois, neighborhoods during the period from 1979 to 1980. Similar to Head Start, the CPC offers comprehensive education and family services. The program is characterized by a collaborative leadership structure, intensive family engagement and parent involvement, low student-staff ratios, health and home visiting services, and a curriculum focused on enhancing school readiness skills in multiple domains. The study also included a demographically matched comparison group with access to the usual early childhood services available for children from low-income families. The intervention and comparison groups were matched on age, intervention eligibility, and socioeconomic status. Intervention eligibility was based on parental consent, residence in a Title I school attendance area, and substantial educational need due to poverty and related factors. See prior work for more in-depth descriptions of the study design and CPC curriculum.^22^ Participants were followed up for 30 years after the end of the intervention. The study sample includes 1083 participants whose educational attainment and ACE data were available by 35 years of age. Data collection procedures were approved by the University of Minnesota’s institutional review board. Informed consent was obtained both orally and in writing; parents provided consent if the participants were younger than 18 years. This cohort study follows the Strengthening the Reporting of Observational Studies in Epidemiology (STROBE) reporting guideline.

### Measures

#### Adverse Childhood Experiences

##### Conventional ACEs

Conventional ACEs are defined as abuse, neglect, and household dysfunction.^1^ Household dysfunction was assessed by retrospective self-report at approximately 35 years of age. Participants were asked if and when a list of events had occurred in their lives. Four of the 5 household dysfunction items from the original ACE studies were used^1^: (1) “You experienced mental illness of a parent or caregiver,” (2) “You were witness to domestic abuse of mother or caregiver,” (3) “One of your parents had problems with alcohol or drugs,” and (4) “One of your parents was arrested by the police during your lifetime.” The fifth household dysfunction ACE of “parent absence or divorce” was excluded given high rates of participants born into single-parent homes (75.4% of the sample [817 of 1083]). Abuse and neglect, the fifth ACE in the present study, was assessed via prospectively collected early childhood child welfare court and Department of Children and Family Services administrative records, supplemented by retrospective self-report. Participants with 1 or more affirmative indicators of any conventional ACEs prior to 5 years of age were coded as 1.

##### Expanded ACEs

The following items were designated as expanded ACEs based on extensive review of adverse life events among youths, as well as prior studies exploring expanded ACE scales^30,37,39,40^: (1) witness to a violent crime, (2) victim of a violent crime, (3) family financial problems, (4) frequent family conflict, (5) death of a parent, (6) death of a sibling, and (7) foster care or out-of-home placement. Items 1 to 6 were based on retrospective self-report, whereas item 7 was based on prospectively collected administrative records. Participants with 1 or more affirmative indicators of any expanded ACEs prior to 5 years of age were coded as 1. A dichotomized indicator was created denoting history of any conventional or expanded ACEs prior to 5 years of age.

#### Covariates

##### CPC Preschool Participation

Students who attended CPC preschool were coded as preschool participants. Child-Parent Center preschool participants comprised 64.3% of the original sample (989 of 1539) and 65.5% of the present study subsample (709 of 1083).

##### CPC School-Age Participation

Students could attend CPC through third grade. Students who attended CPC during early elementary school, whether or not they attended for preschool, were coded as school-aged participants. In this subsample, 57.2% of participants (619 of 1083) overall (71.4% of intervention group participants [506 of 709] and 30.2% of comparison group participants [113 of 374]) had CPC school-aged participation compared with 55.2% (850 of 1539) of the original sample (69.2% of intervention group participants [684 of 989] and 30.2% of comparison group participants [166 of 550]).

##### Sex and Race

Participants were coded 1 if female (50.3% of the original sample [774 of 1539]; 54.9% of the present subsample [594 of 1083]) and 0 if male. Participants were coded 1 if Black (92.9% of the original sample [1430 of 1539]; 93.5% of the present subsample [1013 of 1083]) and 0 if not Black.

##### Demographic Risk Index

The risk index comprises 8 dichotomous indicators measured from birth to 3 years of age: (1) single-parent household, (2) mother younger than 18 years at child’s birth, (3) 4 or more children in household, (4) mother did not complete high school, (5) family income less than 185% of the federal poverty level, (6) mother unemployed, (7) welfare receipt, and (8) residence in a high-poverty neighborhood. The mean (SD) risk index score in this sample was identical to the mean (SD) risk index for the original sample (4.5 [1.7]).

Participants’ sex, race, risk index, CPC preschool participation, and CPC school-aged participation were entered as covariates in analyses examining the associations between ACEs and educational attainment. When examining the interaction between ACEs and preschool attendance, the covariates entered were sex, race, risk index, and CPC school-aged participation.

#### Outcomes: Educational Attainment

Records of attaining a bachelor’s degree or higher or an associate’s degree or higher and the total number of years of education were drawn from National Student Clearinghouse administrative records and interviews at 35 years of age. The measure of an associate’s degree or higher was inclusive of credits beyond an associate’s degree, including individuals with bachelor’s degrees, master’s degrees, and doctoral degrees. The measure of a bachelor’s degree or higher was similarly inclusive of credits beyond a bachelor’s degree.^22^

### Statistical Analysis

Analyses were conducted from July 1 to September 1, 2022. Outcomes were analyzed in Stata, version 14 (StataCorp LLC).^43^ All *P* values were from 2-sided tests, and results were deemed statistically significant at *P* < .05. To correct for potential bias from differential attrition, inverse probability weighting was used for all living participants.^21,22,23,44,45^ Inverse probability weighting uses all available data to estimate complex adjustments independent of the outcome specification model, with weights calculated separately by sex given differential attrition.^46^ See eTable 2 and the eAppendix in Supplement 1 for further detail.

Direct associations of ACEs with educational attainment were estimated using both logistic (for attainment of bachelor’s degree or higher or associate’s degree or higher) and linear (for years of education) regression models. The ACE measures used were conventional ACEs, expanded ACEs, and a measure of either conventional or expanded ACEs, dichotomized to no ACEs or 1 or more ACEs prior to 5 years of age. Participants with no reported ACE exposure (725 of 1083 [66.9%]) served as the reference group in all analyses. Each ACE measure was entered separately with demographic covariates for each education outcome. See eTable 1 in Supplement 1 for list of covariates.

For direct associations of ACEs with the dichotomous outcomes of bachelor’s degree or higher or associate’s degree or higher attainment, odds ratios (ORs) with 95% CIs are presented as standardized measures of the magnitude of the findings. For the magnitude of effect for years of education, mean differences are presented.

For moderation analyses, terms testing the interaction between CPC attendance and ACE exposure were added to the model. Marginal effects indicating the proportion of participants coded as yes for the educational attainment outcome for each condition of ACE exposure and CPC preschool attendance were examined. Odds ratios indicating the magnitude of the difference in the association between ACEs and educational attainment by intervention group are also presented. For standardized measures of the magnitude of moderation findings, effect sizes for the dichotomous outcomes of bachelor’s degree or higher or associate’s degree or higher attainment were calculated based on differences in the proportion of participants with each binary educational attainment outcome by ACEs and then between the intervention and control groups using probit distribution transformations based on marginal effects.

## Results

At the time of 30-year follow-up interview data collection, educational attainment and ACE data were available for 1083 of 1467 living participants (73.8%; mean [SD] age, 35.1 [0.3] years; 594 female participants [54.9%]; 1018 Black participants [94.0%] and 65 Latinx participants [6.0%]).^47^ See eTable 1 in Supplement 1 for full demographic and attrition information.

### Prevalence

A total of 264 participants (24.5%) had 1 or more early conventional ACEs, 199 (18.7%) had 1 or more early expanded ACEs, and 354 (32.8%) had 1 or more early conventional or expanded ACEs (Table 1). Comparison group participants had significantly higher rates of child welfare records in early childhood than CPC participants (7.2% [27 of 374] vs 3.7% [26 of 709]; χ^2^_1,1083_ = 6.6; *P* = .01). By 35 years of age, 214 participants (19.8%) had attained an associate’s degree or higher, and 151 participants (13.9%) had attained a bachelor’s degree or higher. Participants had a mean (SD) of 12.8 (2.2) years of education. The intervention group was significantly more likely than the comparison group to attain both an associate’s degree or higher (22.7% [161 of 709] vs 14.2% [53 of 374]; χ^2^_1,1083_ = 11.3; *P* = .001) and a bachelor’s degree or higher (16.2% [115 of 709] vs 9.6% [36 of 374]; χ^2^_1,1083_ = 8.9; *P* = .003).

**Table 1.** Prevalence of ACEs by Domain and Education in Overall Sample and by Subgroups

ACE	No./total No. (%)			χ^2^ (*df*)	*P* value
	Full sample (N = 1083)	CPC (n = 709)	Comparison (n = 374)		
≥1 Conventional ACEs					
Any	264/1079 (24.5)	167/707 (23.6)	97/372 (26.1)	0.79 (1)	.37
Parent or caregiver mental illness	13/1064 (1.2)	10/704 (1.4)	10/360 (2.8)	0.68 (1)	.41
Domestic violence	62/1059 (5.9)	43/701 (6.1)	19/358 (5.3)	0.29 (1)	.59
Parental substance abuse	162/1064 (15.2)	106/704 (15.1)	56/360 (15.6)	0.05 (1)	.83
Parental arrest	60/1059 (5.7)	39/702 (5.6)	21/357 (5.9)	0.05 (1)	.83
Child welfare record: court and DCFS data	53/1083 (4.9)	26/709 (3.7)	27/374 (7.2)	6.6 (1)	.01
Child welfare record: self-report	38/1061 (3.6)	24/701 (3.4)	14/360 (3.9)	0.15 (1)	.70
Child welfare record: any source	84/1083 (7.8)	47/709 (6.6)	37/374 (9.9)	3.6 (1)	.06
≥1 Expanded ACEs					
Any	199/1067 (18.7)	124/705 (17.6)	75/362 (20.7)	1.5 (1)	.21
Witness to violent crime	18/1062 (1.7)	13/704 (1.9)	5/358 (1.4)	0.29 (1)	.59
Victim of violent crime	6/1063 (0.6)	3/702 (0.4)	3/361 (0.8)	0.69 (1)	.41
Family financial problems	115/1059 (10.9)	69/703 (9.8)	46/356 (12.9)	2.4 (1)	.13
Frequent family conflict	82/1062 (7.7)	61/702 (8.7)	21/360 (5.8)	2.7 (1)	.10
Death of a parent	23/1063 (2.2)	12/704 (1.7)	11/359 (3.1)	2.1 (1)	.15
Death of a sibling	11/1064 (1.0)	9/703 (1.3)	2/361 (0.6)	1.2 (1)	.27
Foster care or out-of-home placement	8/1016 (0.8)	4/665 (0.6)	4/351 (1.1)	0.85 (1)	.36
≥1 Conventional or expanded ACEs	354/1079 (32.8)	219/708 (30.9)	135/371 (36.4)	3.29 (1)	.07
Education outcomes					
≥Associate’s degree	214/1083 (19.8)	161/709 (22.7)	53/374 (14.2)	11.3 (1)	.001
≥Bachelor’s degree	151/1083 (13.9)	115/709 (16.2)	36/374 (9.6)	8.9 (1)	.003
Years of education, No. (mean [SD])	1082 (12.8 [2.2])	709 (13.0 [2.2])	373 (12.4 [2.0])	Mean difference, −0.69 (95% CI, −0.96 to −0.42)	<.001

Abbreviations: ACEs, adverse childhood experiences; CPC, Child-Parent Center; DCFS, Department of Children and Family Services.

### Direct Associations Between ACEs and Educational Attainment

ACEs were not associated with educational attainment in the full sample or in the CPC intervention subgroup. However, both the dichotomized measure of conventional or expanded ACEs and conventional ACEs alone were associated with reduced educational attainment across outcomes for the comparison group (Table 2). Comparison group participants with either conventional or expanded ACEs were approximately one-fourth as likely as comparison group participants without ACEs to attain a bachelor’s degree or higher (OR, 0.26; 95% CI, 0.09-0.70) or an associate’s degree or higher (OR, 0.26; 95% CI, 0.11-0.62). Comparison group participants with either type of ACEs also completed less school overall than those in the comparison group without early ACEs (β = −0.64; 95% CI, −1.02 to −0.26). A direct association of expanded ACEs was seen only for the years of education outcome (β = −0.53; 95% CI, −1.01 to −0.04).

**Table 2.** Associations Between ACEs and Educational Attainment, in Overall Sample and by Subgroups

Outcome	Full sample			CPC intervention group			Comparison group		
	ACEs-C	ACEs-E	ACEs-CE	ACEs-C	ACEs-E	ACEs-CE	ACEs-C	ACEs-E	ACEs-CE
**Bachelor’s degree or higher**									
No.	990	925	1080	656	613	708	331	309	369
OR (95% CI)^a^	0.75 (0.48 to 1.18)	0.92 (0.58 to 1.47)	0.84 (0.56 to 1.25)	1.04 (0.63 to 1.71)	1.20 (0.71 to 2.03)	1.16 (0.74 to 1.82)	0.21 (0.06 to 0.71)	0.39 (0.14 to 1.14)	0.26 (0.09 to 0.70)
**Associate’s degree or higher**									
No.	990	925	1080	656	613	708	331	309	369
OR (95% CI)^a^	0.69 (0.46 to 1.02)	0.97 (0.64 to 1.46)	0.82 (0.58 to 1.17)	0.97 (0.62 to 1.52)	1.28 (0.79 to 2.07)	1.15 (0.77 to 1.72)	0.16 (0.05 to 0.48)	0.44 (0.17 to 1.14)	0.26 (0.11 to 0.62)
**Years of education**									
No.	989	924	1079	656	705	708	332	310	370
β (95% CI)^a^	−0.29 (−0.59 to 0.00)	−0.12 (−0.45 to 0.22)	−0.19 (−0.46 to 0.07)	−0.10 (−0.50 to 0.31)	0.13 (−0.31 to 0.58)	0.07 (−0.28 to 0.43)	−0.68 (−1.10 to −0.28)	−0.53 (−1.01 to −0.04)	−0.64 (−1.02 to −0.26)
Mean difference	−0.20	−0.12	−0.13	−0.07	0.09	0.05	0.51	0.39	0.48

Abbreviations: ACEs, adverse childhood experiences; ACEs-C, conventional adverse childhood experiences; ACEs-CE, conventional or expanded adverse childhood experiences; ACEs-E, expanded adverse childhood experiences; CPC, Child-Parent Center; OR, odds ratio.

^a^ The 0 ACE group serves as the reference group for all analyses.

### Interaction Between ACEs and Preschool Attendance on Educational Attainment

**Figure 1.**
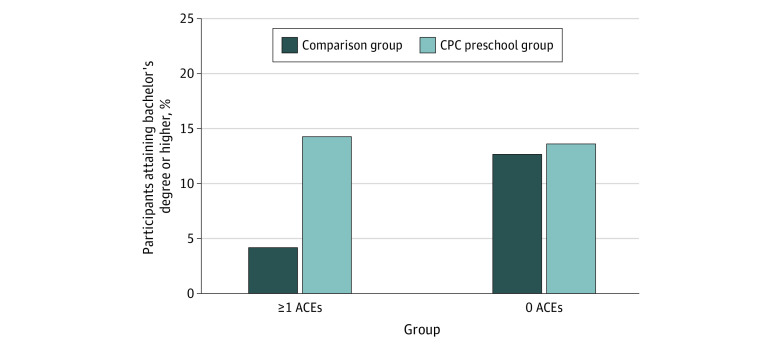
Percentage of Participants Attaining a Bachelor’s Degree or Higher by Adverse Childhood Experience (ACE) and Intervention Status CPC indicates Child-Parent Center.

**Figure 2.**
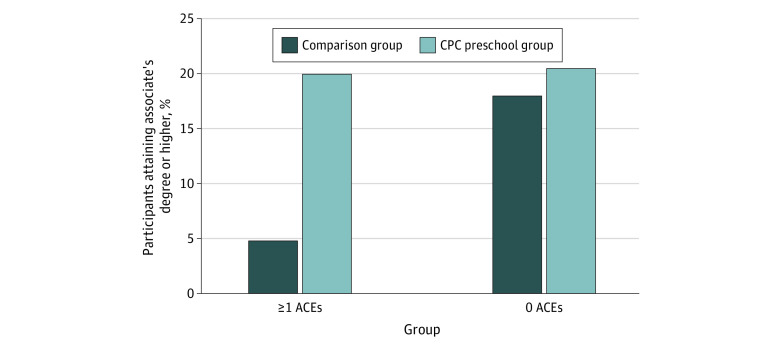
Percentage of Participants Attaining an Associate’s Degree or Higher by Adverse Childhood Experience (ACE) and Intervention Status CPC indicates Child-Parent Center.

Overall, for participants with both early conventional and expanded ACEs, CPC preschool attendance evinced a compensatory association with educational attainment. Participants who attended CPC preschool completed similar rates of education as CPC participants without early ACEs (Table 3, Figure 1, and Figure 2).

**Table 3.** Marginal Effects for Interaction Between ACEs and CPC Preschool Attendance

Group	Participants with BA or higher, %	Group difference (standardized difference), %^a^	Participants with AA or higher, %	Group difference (standardized difference), %^a^	Years of education	Group difference (SD), y
Conventional ACEs						
Total No.	990	NA	990	NA	989	NA
≥1 ACEs, CPC preschool	14.3	0.6 (0.03)	20.3	0.1 (0.0)	12.8	−0.1 (−0.04)
No ACEs, CPC preschool	13.7		20.2		12.9	
≥1 ACEs, comparison group	3.3	−8.9 (−0.7)	3.9	−13.4 (−0.6)	11.9	−0.6 (−0.3)
No ACEs, comparison group	12.2		17.3		12.5	
Summary of group differences	NA	−9.5 (−0.7)	NA	−13.5 (−0.6)	NA	NA
OR (95% CI)^b^	4.58 (1.28 to 16.39)		5.64 (1.84 to 17.29)		0.56 (0.00 to 1.13)^b^	
*P* value	.02		.002		.05	
Expanded ACEs						
Total No.	924	NA	924	NA	923	NA
≥1 ACEs, CPC preschool	16.4	2.7 (0.1)	24.5	4.5 (0.2)	13.0	0.2 (0.1)
No ACEs, CPC preschool	13.7		20.0		12.8	
≥1 ACEs, comparison group	5.3	−7.4 (−0.5)	8.9	−9.1 (−0.4)	12.0	−0.1 (−0.3)
No ACEs, comparison group	12.7		18.0		13.0	
Summary of group differences	NA	−10.1 (−0.6)	NA	−13.6 (−0.6)	NA	NA
OR (95% CI)^b^	3.42 (1.07 to 10.99)		3.16 (1.18 to 8.49)		0.72 (0.06 to 1.38)^b^	
*P* value	.04		.02		.03	
Conventional or expanded ACEs						
Total No.	1079	NA	1079	NA	1078	NA
≥1 ACEs, CPC preschool	15.4	1.8 (0.04)	22.4	2.5 (0.1)	12.9	0.1 (0.03)
No ACEs, CPC preschool	13.6		19.9		12.8	
≥1 ACEs, comparison group	3.7	−8.4 (−0.6)	5.6	−11.5 (−0.7)	11.9	−0.6 (−0.3)
No ACEs, comparison group	12.1		17.1		12.5	
Summary of group differences	NA	−10.2 (−0.7)	NA	−14.0 (−0.7)	NA	−0.71 (−0.35)
OR (95% CI)^b^	4.44 (1.56 to 12.68)		4.39 (1.81 to 10.64)		0.72 (0.20 to 1.24)^c^	
*P* value	.005		.001		.007	

Abbreviations: AA, associate’s degree; ACEs, adverse childhood experiences; BA, bachelor’s degree; CPC, Child-Parent Center; NA, not applicable; OR, odds ratio.

^a^ The standardized difference for proportions was based on the probit transformation of percentages.

^b^ The 0 ACE group serves as the reference group for all analyses.

^c^ β Coefficient (95% CI).

#### Combined Conventional and Expanded ACEs

##### Bachelor’s Degree or Higher

The interaction between conventional or expanded ACEs and CPC preschool was associated with attainment of a bachelor’s degree or higher (OR, 4.44; 95% CI, 1.56-12.68; *P* = .005). Participants with either conventional or expanded ACEs who attended CPC preschool attained a bachelor’s degree or higher at a similar rate as attendees without ACEs (15.4% vs 13.6%; difference, 1.8 percentage points), while comparison group participants with ACEs had lower rates of attainment of a bachelor’s degree or higher than those without ACEs (3.7% vs 12.1%; difference, −8.4 percentage points) (Figure 1).

##### Associate’s Degree or Higher

A significant interaction between conventional or expanded ACEs and CPC preschool was also associated with attainment of an associate’s degree or higher (OR, 4.39; 95% CI, 1.81-10.64; *P* = .001). Participants with 1 or more conventional or expanded ACEs who attended CPC preschool attained an associate’s degree or higher at similar rates to those without ACEs (22.4% vs 19.9%; difference, 2.5 percentage points). For participants in the comparison group, those with ACEs attained an associate’s degree or higher at lower rates than those without ACEs (5.6% vs 17.1%; difference, −11.5 percentage points) (Figure 2).

##### Years of Education

The interaction between conventional or expanded ACEs and CPC preschool was associated with years of education completed (β = 0.72; 95% CI, 0.20-1.24; *P* = .007). Among the participants in the intervention group, those with 1 or more conventional or expanded ACEs completed similar years of education as those without ACEs (12.9 vs 12.8 years; difference, 0.07 years). In the comparison group, participants with ACEs completed over half a year less of education than those without ACEs (11.9 vs 12.5 years; difference, −0.64 years).

#### Conventional ACEs

The interactions between conventional ACEs and CPC participation for attaining a bachelor’s degree or higher and an associate’s degree or higher (Figure 1) were significant, with similar patterns of results as those seen for the measure of conventional or expanded ACEs (OR, 4.58; 95% CI, 1.28-16.39; *P* = .02; and OR, 5.64; 95% CI, 1.84-17.29; *P* = .002, respectively) (Table 3). The association of the interaction between conventional ACEs and CPC attendance with years of education was not statistically significant.

#### Expanded ACEs

Interactions between expanded ACEs and CPC were significant across all 3 outcomes, with participants who had early expanded ACEs and attended CPC preschool achieving rates of educational attainment similar to or higher than their counterparts without ACEs, but with participants in the comparison group with early expanded ACEs demonstrating significantly lower educational attainment than their peers without ACEs (bachelor’s degree or higher: OR, 3.42; 95% CI, 1.07-10.99; *P* = .04; associate’s degree or higher: OR, 3.16; 95% CI, 1.18-8.49; *P* = .02; years of education: β = 0.72; 95% CI, 0.06-1.38; *P* = .03; Table 3).

## Discussion

The findings of this study suggest that ACEs are associated with reduced educational attainment and that comprehensive ECE may compensate for these associations. Child-Parent Center preschool intervention attendees who had exposure to either conventional or expanded ACEs in early childhood achieved equivalent educational attainment as their counterparts not exposed to ACEs, while youths in the comparison group exposed to ACEs attained postsecondary education at lower rates than their peers without ACEs. These results contribute to the research base suggesting that youths at higher risk may benefit most from intervention and provide support for including ACEs as a standard indicator of risk.

A crossover interaction was observed for early expanded ACEs and attainment of an associate’s degree or higher and a bachelor’s degree or higher, wherein the group of participants exposed to ACEs who were in the CPC intervention seemed to achieve higher educational attainment than intervention participants without ACEs. It is possible that children with exposure to only expanded ACEs were able to benefit more from the intensive enrichment environment provided by CPC, but further research is needed to understand the mechanisms of this connection.

These findings have clear implications for policy and practice. As evidence conclusively points to serious long-term implications of ACEs, measuring and identifying ways to mitigate these implications are crucial. Comprehensive ECE programs such as CPC preschool, which continues to serve thousands of children in the Midwestern United States, are one such mechanism. Decades of developmental science tells us that opportunities to repair and intervene are as numerous as consequences of adversity, but evidence also shows that such interventions are far more effective when started early.^48^ In the present study, ECE was positively associated with educational attainment for youths with the most challenging early environments, which has important implications for socioeconomic well-being and health. This study suggests that when assessing risk, clinicians and child-facing practitioners should consider not only poverty and maltreatment but also ACEs more broadly. Clinicians should screen for the presence of early ACEs so that they may refer families to comprehensive ECE and other services.

### Strengths and Limitations

This study has several strengths, including the use of a large longitudinal sample with a retention rate of approximately 70% and prospective collection of maltreatment and education information via administrative data. In addition, few large-scale ACE studies have examined the association of ACEs with education, particularly in a population of primarily Black participants. A recent scoping review found that only 6.6% of nearly 1500 articles investigating childhood adversity discussed protective factors “beyond the individual or family level.”^49^ The authors recommended that “Future ACEs research be incorporated into broader, strengths-based and action-oriented frameworks focused on social determinants of health and health inequities.”^49^^(p2)^ The present study is consistent with this expansion of focus to actionable protective factors.

This study also has several limitations. All ACEs, with the exception of those from prospectively collected records of child welfare involvement and out-of-home placement, were retrospectively reported. Moreover, the prospective indicator of maltreatment was a dichotomous variable indicating child welfare involvement broadly, as court and Department of Children and Family Services records did not differentiate the type of early childhood maltreatment. Underreporting of all forms of maltreatment is common, particularly for young children. Actual rates are likely higher than reported. Although much prior ACE research has used polychotomized ACE scores (1, 2, 3, and ≥4), given our focus on a truncated time period (≤5 years as opposed to ≤18 years), fewer than 10% of participants had 2 or more conventional or expanded ACEs. As such, the dichotomized indicator was deemed most appropriate. Finally, many participants also reported on a partial list of ACEs at 24 years of age. The self-report at 35 years of age was used for the present study due to the addition of crucial ACE items. Prior consistency analyses of participants who responded to the items asked at both time points indicated that more than 80% of the sample responded consistently to 6 or more of 8 items.^41^

## Conclusions

In this cohort study of 1083 Chicago Longitudinal Study participants followed up for more than 30 years, results were consistent with prior evidence that ECE can be an avenue for promoting economic well-being, particularly for children at the highest risk. Moreover, findings suggested that conceptualizations of risk should explicitly include both conventional and expanded ACE exposure. Although experiencing ACEs early in life can reduce educational attainment, CPC preschool may compensate for this outcome. Comprehensive ECE programs, such as CPC, can foster resilience and mitigate the effects of early adversity. Such interventions are an established, evidence-based, scalable tool to level the playing field for the most vulnerable children.

## Notes

Supplement 1. **eTable 1.** Chicago Longitudinal Study (CLS) Age 35 Follow-up and Attrition Sample on Child and Family Background Attributes **eTable 2.** Predictor Variables for Age 35 Sample Retention in Sex-Split IPW Model (N=1,531-1,539)
eAppendix.

eReferences.

Supplement 2.
Data Sharing Statement

## References

[1] Felitti VJ, Anda RF, Nordenberg D, . Relationship of childhood abuse and household dysfunction to many of the leading causes of death in adults: the Adverse Childhood Experiences (ACE) Study. Am J Prev Med. 1998;14(4):245-258. doi:10.1016/S0749-3797(98)00017-8 9635069

[2] Merrick MT, Ports KA, Ford DC, Afifi TO, Gershoff ET, Grogan-Kaylor A. Unpacking the impact of adverse childhood experiences on adult mental health. Child Abuse Negl. 2017;69:10-19. doi:10.1016/j.chiabu.2017.03.016 28419887 PMC6007802

[3] Sheffler JL, Stanley I, Sachs-Ericsson N. ACEs and mental health outcomes. In: Asmundson GJG, Afifi TO, eds. Adverse Childhood Experiences: Using Evidence to Advance Research, Practice, Policy, and Prevention. Elsevier Academic Press; 2020.

[4] Blodgett C, Lanigan JD. The association between adverse childhood experience (ACE) and school success in elementary school children. Sch Psychol Q. 2018;33(1):137-146. doi:10.1037/spq0000256 29629790

[5] Hinojosa R, Nguyen J, Sellers K, Elassar H. Barriers to college success among students that experienced adverse childhood events. J Am Coll Health. 2019;67(6):531-540. doi:10.1080/07448481.2018.1498851 30230975

[6] Jimenez ME, Wade R Jr, Lin Y, Morrow LM, Reichman NE. Adverse experiences in early childhood and kindergarten outcomes. Pediatrics. 2016;137(2):e20151839. doi:10.1542/peds.2015-1839 26768347 PMC4732356

[7] Giovanelli A, Reynolds AJ, Mondi CF, Ou SR. Adverse childhood experiences and adult well-being in a low-income, urban cohort. Pediatrics. 2016;137(4):e20154016. doi:10.1542/peds.2015-4016 26966132 PMC4991352

[8] Hardcastle K, Bellis MA, Ford K, Hughes K, Garner J, Ramos Rodriguez G. Measuring the relationships between adverse childhood experiences and educational and employment success in England and Wales: findings from a retrospective study. Public Health. 2018;165:106-116. doi:10.1016/j.puhe.2018.09.014 30388488

[9] Haskins R, Holzer H, Lerman R. Promoting economic mobility by increasing postsecondary education. Economic Mobility Project. Accessed July 28, 2022. https://webarchive.urban.org/uploadedpdf/1001280_promotingeconomic.pdf

[10] Carlson RH, McChesney CS. Income sustainability through educational attainment. J Educ Train Stand. 2015;3(1):108-115. doi:10.11114/jets.v3i1.508

[11] Heckman JJ, Lochner LJ, Todd PE. Earnings functions and rates of return. J Hum Cap. 2008;2:1-31. doi:10.1086/587037

[12] Congressional Research Service. Real Wage Trends, 1979 to 2018. Congressional Research Service; 2019.

[13] Smeeding TM. Multiple barriers to economic opportunity for the “truly” disadvantaged and vulnerable. RSF. 2016;2(2):98-122. doi:10.7758/rsf.2016.2.2.04 30123833 PMC6095670

[14] Sabol TJ, Sommer TE, Chase-Lansdale PL, Brooks-Gunn J. Intergenerational economic mobility for low-income parents and their children: a dual developmental science framework. Annu Rev Psychol. 2021;72:265-292. doi:10.1146/annurev-psych-010419-051001 32966174

[15] Mazumder B. Black-White differences in intergenerational economic mobility in the United States. Econ Perspect. 2014;38(1):1-18. Accessed May 10, 2023. https://ssrn.com/abstract=2434178

[16] Campbell FA, Pan Y, Burchinal M. Sustaining gains from early childhood intervention: the Abecedarian program. In: Reynolds AJ, Temple JA, eds. Sustaining Early Childhood Learning Gains: Program, School, and Family Influences. Cambridge University; 2019. doi:10.1017/9781108349352.013

[17] Heckman JJ. Skill formation and the economics of investing in disadvantaged children. Science. 2006;312(5782):1900-1902. doi:10.1126/science.1128898 16809525

[18] Schweinhart LJ. 11 Lessons on sustaining early gains from the life-course study of Perry Preschool. In: Reynolds AJ, Temple JA, eds. Sustaining Early Childhood Learning Gains: Program, School, and Family Influences. Cambridge University; 2019. doi:10.1017/9781108349352.012

[19] Karoly LA. The economic returns to early childhood education. Future Child. 2016;26:37-55. doi:10.1353/foc.2016.0011

[20] Reynolds AJ, Temple JA, White BA, Ou SR, Robertson DL. Age 26 cost-benefit analysis of the Child-Parent Center early education program. Child Dev. 2011;82(1):379-404. doi:10.1111/j.1467-8624.2010.01563.x 21291448 PMC3817956

[21] Reynolds AJ, Ou SR, Mondi CF, Giovanelli A. Reducing poverty and inequality through preschool-to-third-grade prevention services. Am Psychol. 2019;74(6):653-672. doi:10.1037/amp0000537 31545639 PMC6767908

[22] Reynolds AJ, Ou SR, Temple JA. A multicomponent, preschool to third grade preventive intervention and educational attainment at 35 years of age. JAMA Pediatr. 2018;172(3):247-256. doi:10.1001/jamapediatrics.2017.4673 29379955 PMC5885840

[23] Reynolds AJ, Temple JA, Ou SR, Arteaga IA, White BAB. School-based early childhood education and age-28 well-being: effects by timing, dosage, and subgroups. Science. 2011;333(6040):360-364. doi:10.1126/science.1203618 21659565 PMC3774305

[24] Anda RF, Butchart A, Felitti VJ, Brown DW. Building a framework for global surveillance of the public health implications of adverse childhood experiences. Am J Prev Med. 2010;39(1):93-98. doi:10.1016/j.amepre.2010.03.015 20547282

[25] Odgers CL, Jaffee SR. Routine versus catastrophic influences on the developing child. Annu Rev Public Health. 2013;34:29-48. doi:10.1146/annurev-publhealth-031912-114447 23297656 PMC4212823

[26] Walsh D, McCartney G, Smith M, Armour G. Relationship between childhood socioeconomic position and adverse childhood experiences (ACEs): a systematic review. J Epidemiol Community Health. 2019;73(12):1087-1093. doi:10.1136/jech-2019-212738 31563897 PMC6872440

[27] Fass S, Dinan KA, Aratani Y. Child poverty and intergenerational mobility. National Center for Children in Poverty. Accessed July 28, 2022. http://www.nccp.org/wp-content/uploads/2009/12/text_911.pdf

[28] Sacks V, Murphey D. The prevalence of adverse childhood experiences, nationally, by state, and by race or ethnicity. ChildTrends. Accessed July 28, 2022. https://www.childtrends.org/publications/prevalence-adverse-childhood-experiences-nationally-state-race-ethnicity

[29] Choi JK, Wang D, Jackson AP. Adverse experiences in early childhood and their longitudinal impact on later behavioral problems of children living in poverty. Child Abuse Negl. 2019;98:104181. doi:10.1016/j.chiabu.2019.104181 31521904

[30] Cronholm PF, Forke CM, Wade R Jr, . Adverse childhood experiences: expanding the concept of adversity. Am J Prev Med. 2015;49(3):354-361. doi:10.1016/j.amepre.2015.02.001 26296440

[31] Richards TN, Schwartz JA, Wright E. Examining adverse childhood experiences among Native American persons in a nationally representative sample: differences among racial/ethnic groups and race/ethnicity–sex dyads. Child Abuse Negl. 2021;111:104812. doi:10.1016/j.chiabu.2020.104812 33220946

[32] Wade R Jr, Cronholm PF, Fein JA, . Household and community-level adverse childhood experiences and adult health outcomes in a diverse urban population. Child Abuse Negl. 2016;52:135-145. doi:10.1016/j.chiabu.2015.11.021 26726759

[33] Child Welfare Information Gateway. Rural child welfare practice. Accessed July 28, 2022. https://www.childwelfare.gov/pubpdfs/rural.pdf

[34] Sheats KJ, Irving SM, Mercy JA, . Violence-related disparities experienced by Black youth and young adults: opportunities for prevention. Am J Prev Med. 2018;55(4):462-469. doi:10.1016/j.amepre.2018.05.017 30139709 PMC6691967

[35] Milner J, Kelly D. It’s time to stop confusing poverty with neglect. *The Imprint*. Accessed July 28, 2022. https://imprintnews.org/child-welfare-2/time-for-child-welfare-system-to-stop-confusing-poverty-with-neglect/40222

[36] Wulczyn F, Lery B. Racial disparity in foster care admissions. Chapin Hall Center for Children at the University of Chicago. Accessed July 28, 2022. https://citizenreviewpanelsny.org/documents/chapin_hall_document.pdf

[37] Choi C, Mersky JP, Janczewski CE, Lee CTP, Davies WH, Lang AC. Validity of an expanded assessment of adverse childhood experiences: a replication study. Child Youth Serv Rev. 2020;117:105216. doi:10.1016/j.childyouth.2020.105216

[38] Finkelhor D, Turner HA, Shattuck A, Hamby SL. Prevalence of childhood exposure to violence, crime, and abuse: results from the National Survey of Children’s Exposure to Violence. JAMA Pediatr. 2015;169(8):746-754. doi:10.1001/jamapediatrics.2015.0676 26121291

[39] Karatekin C, Hill M. Expanding the original definition of adverse childhood experiences (ACEs). J Child Adolesc Trauma. 2018;12(3):289-306. doi:10.1007/s40653-018-0237-5 32318200 PMC7163861

[40] Finkelhor D, Shattuck A, Turner H, Hamby S. Improving the adverse childhood experiences study scale. JAMA Pediatr. 2013;167(1):70-75. doi:10.1001/jamapediatrics.2013.420 23403625

[41] Giovanelli A, Reynolds AJ. Adverse childhood experiences: the importance of context. Prev Med. 2021;148:106557. doi:10.1016/j.ypmed.2021.106557 33857559 PMC8594423

[42] Giovanelli A, Mondi CF, Reynolds AJ, Ou SR. Adverse childhood experiences: mechanisms of risk and resilience in a longitudinal urban cohort. Dev Psychopathol. 2020;32(4):1418-1439. doi:10.1017/S095457941900138X 31663487 PMC7190431

[43] StataCorp. Stata: release 17. StataCorp LP; 2021.

[44] Mondi CF, Reynolds AJ. Psychological wellbeing in early midlife following early childhood intervention. Dev Psychopathol. 2022;24:1-26. doi:10.1017/S0954579421001528 35068402 PMC9308829

[45] Mondi CF, Reynolds AJ, Richardson B. Early childhood educational intervention and depressive symptoms in emerging adulthood: an inverse probability weighting analysis. Eval Rev. 2020;44(5-6):379-409. doi:10.1177/0193841X20976527 33307776 PMC8127666

[46] Seaman SR, White IR. Review of inverse probability weighting for dealing with missing data. Stat Methods Med Res. 2013;22(3):278-295. doi:10.1177/0962280210395740 21220355

[47] Ou SR, Mondi CF, Yoo S, Park K, Warren B, Reynolds AJ. Thirty years later: locating and interviewing participants of the Chicago Longitudinal Study. Early Child Res Q. 2020;51:1-13. doi:10.1016/j.ecresq.2019.08.002 31933509 PMC6957089

[48] Zeanah CH, Gunnar MR, McCall RB, Kreppner JM, Fox NA. Sensitive periods. Monogr Soc Res Child. 2011;76(4):147-162. doi:10.1111/j.1540-5834.2011.00631.x PMC4130246 25125708

[49] Karatekin C, Mason SM, Riegelman A, . Adverse childhood experiences: a scoping review of measures and methods. Child Youth Serv Rev. 2022;136:1-26. doi:10.1016/j.childyouth.2022.106425

