# Supplementary material for: Evaluation of Midlife Educational Attainment Among Attendees of a Comprehensive Early Childhood Education Program in the Context of Early Adverse Childhood Experiences
Source: JAMA Netw Open. 2023 Jun 22;6(6):e2319372. doi: 10.1001/jamanetworkopen.2023.19372 (PMC10288333; doi:10.1001/jamanetworkopen.2023.19372)
Supplement: Supplement 1. — eTable 1. Chicago Longitudinal Study (CLS) Age 35 Follow-up and Attrition Sample on Child and Family Background Attributes eTable 2. Predictor Variables for Age 35 Sample Retention in Sex-Split IPW Model (N=1,531-1,539) eAppendix. eReferences. [file jamanetwopen-e2319372-s001.pdf]

Supplementary Online Content

Giovanelli A, Mondi CF, Reynolds AJ, Ou SR. Evaluation of midlife educational attainment among attendees of a comprehensive early childhood education program in the context of early adverse childhood experiences. *JAMA Netw Open.* 2023;6(6):e2319372. doi:10.1001/jamanetworkopen.2023.19372

eTable 1. Chicago Longitudinal Study (CLS) Age 35 Follow-up and Attrition Sample on Child and Family Background Attributes
eTable 2. Predictor Variables for Age 35 Sample Retention in Sex-Split IPW Model (N=1,531-1,539)
eAppendix.
eReferences.

This supplementary material has been provided by the authors to give readers additional information about their work.

© 2023 Giovanelli A et al. *JAMA Network Open*.

eTable 1. Chicago Longitudinal Study (CLS) Age 35 Follow-up and Attrition Sample on Child and Family Background Attributes

	Attrition sample (*n* = 456)[a]	Study sample (*n* = 1083)	Mean difference	95% confidence interval of difference	
				Lower	Upper
Female	0.39	0.55	.16	.11	.21
Black	0.92	0.94	.02	-0.01	0.05
Participation in the CPC program in preschool	0.61	0.65	.04	-0.01	0.09
Participation in CPC program beyond preschool	0.51	0.57	.06	0.01	0.12
Low birthweight (<2500 grams)	0.09	0.14	.04	0.01	0.08
Demographic Risk Index, child age 0-3	4.67	4.46	-.21	-0.40	-0.03
Mother under age 18 years at child's birth	0.18	0.15	-.03	-0.07	0.01
Mother did not complete high school	0.57	0.53	-.05	-0.10	0.01
Mother years of education (continuous)	10.98	11.15	.17	-0.03	0.36
Child in single-parent household	0.79	0.75	-.04	-0.08	0.01
Four or more children in household	0.16	0.17	.01	-0.03	0.05
Participation in public aid (AFDC)	0.67	0.61	-.06	-0.11	0.00
Child eligible for free school lunch	0.86	0.83	-.03	-0.07	0.02
Mother not employed full- or part-time	0.68	0.66	-.03	-0.08	0.03
Neighborhood poverty	0.77	0.76	-.01	-0.06	0.04

All variables are dichotomous except for mother years of education and eight-item demographic risk index. CPC = Child-Parent Center. AFDC = Aid for Families with Dependent Children.

© 2023 Giovanelli A et al. *JAMA Network Open.*

eTable 2. Predictor Variables for Age 35 Sample Retention in Sex-Split IPW Model (N=1,531-1,539)

Variable #	Variable description	Mean (*S.D.*)	% of sample (dichotomous variables)
1	CPC preschool participation	--	64.30
2	School-age CPC participation	--	55.20
3	African American	--	93.00
4	Low birth weight	--	11.80
5	Word analysis skills at the end of kindergarten	63.82 (13.25)	--
6	Composite school readiness score	47.37 (8.77)	--
7	Substantiated maltreatment between ages zero to three years	--	3.80
8	Mother was not a high school graduate by participant age three	--	54.30
9	Eligible for free lunch between ages zero and three	--	83.80
10	Mother was under age 18 at the participant's birth	--	16.20
11	Lived in a household of four or more children between ages zero and three	--	16.60
12	Family income was below 185% of the federal poverty level between ages zero and three	--	62.80
13	Mother was unemployed or employed part-time when participant was between ages zero and three	--	66.30
14	Lived in a single parent household between ages zero and three	--	76.50
15	Information was not available about age zero to three risk indicators (items 9-15 above)	--	16.20
16	Lived in a school attendance area where at least 60% of households were impoverished	--	76.00
17	Interaction term: CPC preschool * age 0-3 sociodemographic risk index	2.90 (2.54)	--
18-21	Percentage of individuals living one year within the participants' housing unit by age four	0.19 (0.07)	--
19	Percentage of individuals living between one and five years within the participants' housing unit by age four	0.29 (0.07)	--
20	Percentage of individuals living five to ten years within the participants' housing unit by age four	0.23 (0.09)	--
21	Percentage of individuals living ten to twenty years within the participants' housing unit by age four	0.25 (0.11)	--
22	Percentage of self-employed individuals ages 16 and older within the participant's census tract by age four	0.02 (0.02)	--
23	Percentage of female-headed black households within the participant's census tract by age four	0.40 (0.15)	--
24	Frequent family conflict between ages zero and five	--	5.70
25	Family financial problems between ages zero and five	--	7.00
26	Parental substance abuse problems between ages zero and five	--	4.10
27	Socio-demographic risk index (age eight)	4.25 (1.79)	--
28	Socio-demographic risk index (age 12)	4.23 (1.82)	--
29	Grades 1-3 teacher-rated socioemotional functioning	19.14 (4.67)	--
30	Grades 4-6 teacher-rated socioemotional functioning	18.54 (4.45)	--
31	Percentage of birth tract population, ages 25+ years, with four or more years of college attendance in 1990	5.75 (6.47)	--
32	Number of years of magnet school attendance between fourth and eighth grade	0.40 (1.29)	--
33	Eighth grade reading score	145.0 (20.74)	--
34	Number of years active in Chicago Public Schools between kindergarten and 12th grade	9.04 (3.54)	--
35	Number of school moves between kindergarten and 12th grade	3.04 (1.50)	--
36	Dropped out of high school before age 16	--	12.5
37	Graduated from high school on-time	--	37.8
38	Socio-demographic risk index (age 17)	3.74 (1.48)	--
39	Ever arrested before age 18	--	30.6

© 2023 Giovanelli A et al. *JAMA Network Open.*

40	Number of felony arrests by age 26	0.48 (1.15)	--
41	Socioeconomic Status Index (ages 24-27) - includes average annual earnings from 2004-2007 and educational attainment by age 26.	2.99 (2.11)	--
42	"Stable employment" (ages 24-27) - 8 or more quarters with any earnings from 2004-2007.	--	45.8
43	Average annual earnings (ages 24-27).	1.13 (1.30)	--
44	Moderate or higher occupational prestige/skill (age 24-27).	--	23.9
45	Highest grade completed by August 2008	12.06 (1.65)	--
46	Number of missing adult outcomes (items 41-46)	4.17 (2.38)	--
47	CLS located a Social Security Number by 2007	--	93.5
48	Number of years received TANF (ages 24-27).	0.10 (0.40)	--
49	Last known address as of July 2017 was in Illinois	--	76.9

© 2023 Giovanelli A et al. *JAMA Network Open.*

eAppendix.

The IPW process entails conditioning a logistic regression on a set of predictors (X) hypothesized to influence participants' probabilities of sample retention (no attrition) at follow-up (Y). Missing data on predictors and covariates (which ranged from ~5-20%) were imputed using the expectation–maximization algorithm. In this study, IPW methods yielded predicted probabilities of living CLS participants being successfully recruited to participate in the follow-up survey.

Weights were calculated separately by sex given differential attrition.[1] Consistent with prior research,[2-6] a comprehensive set of variables was included in the model predicting probability of being in the follow-up sample. Post-IPW model fit was determined by examining the balance of covariates between the two groups.[7] The estimated CPC program effect is represented by between-group differences in predicted probabilities for sample retention.

eReferences.

1. Seaman SR, White IR. Review of inverse probability weighting for dealing with missing data. Stat Methods Med Res. 2013;22(3):278-295. Medline:21220355 doi:10.1177/0962280210395740
2. Reynolds AJ, Ou SR, Mondi CF, Giovanelli A. Reducing poverty and inequality through preschool-to-third-grade prevention services. Am Psychol. 2019;74(6):653-672. Medline:31545639 doi:10.1037/amp0000537
3. Reynolds AJ, Ou SR, Temple JA. A multicomponent, preschool to third grade preventive intervention and educational attainment at 35 years of age. JAMA Pediatr. 2018;172(3):247-256. Medline:29379955 doi:10.1001/jamapediatrics.2017.4673
4. Reynolds AJ, Temple JA, Ou SR, Arteaga IA, White BAB. School-based early childhood education and age-28 well-being: effects by timing, dosage, and subgroups. Science. 2011;333(6040):360-364. Medline:21659565 doi:10.1126/science.1203618
5. Mondi CF, Reynolds AJ. Psychological wellbeing in early midlife following early childhood intervention. Dev Psychopathol. 2022;24:1-26. Medline:35068402
6. Mondi CF, Reynolds AJ, Richardson B. Early childhood educational intervention and depressive symptoms in emerging adulthood: an inverse probability weighting analysis. Eval Rev. 2020;44(6-6):379-409. Medline:33307776 doi:10.1177/0193841X20976527
7. Li F, Morgan KL, Zaslavsky AM. Balancing covariates via propensity score weighting. J Am Stat Assoc. 2018;113:390-400. doi:10.1080/01621459.2016.1260466

© 2023 Giovanelli A et al. *JAMA Network Open.*
