# Supplementary material for: Evaluation of Midlife Educational Attainment Among Attendees of a Comprehensive Early Childhood Education Program in the Context of Early Adverse Childhood Experiences
Source: JAMA Netw Open. 2023 Jun 22;6(6):e2319372. doi: 10.1001/jamanetworkopen.2023.19372 (PMC10288333; doi:10.1001/jamanetworkopen.2023.19372)
Supplement: Supplement 2. — Data Sharing Statement [file jamanetwopen-e2319372-s002.pdf]

Data Sharing Statement

Giovanelli. Evaluation of Midlife Educational Attainment Among Attendees of a Comprehensive Early Childhood Education Program in the Context of Early Adverse Childhood Experiences. *JAMA Netw Open*. Published June 22, 2023. doi:10.1001/jamanetworkopen.2023.19372

Data
Data available: No
